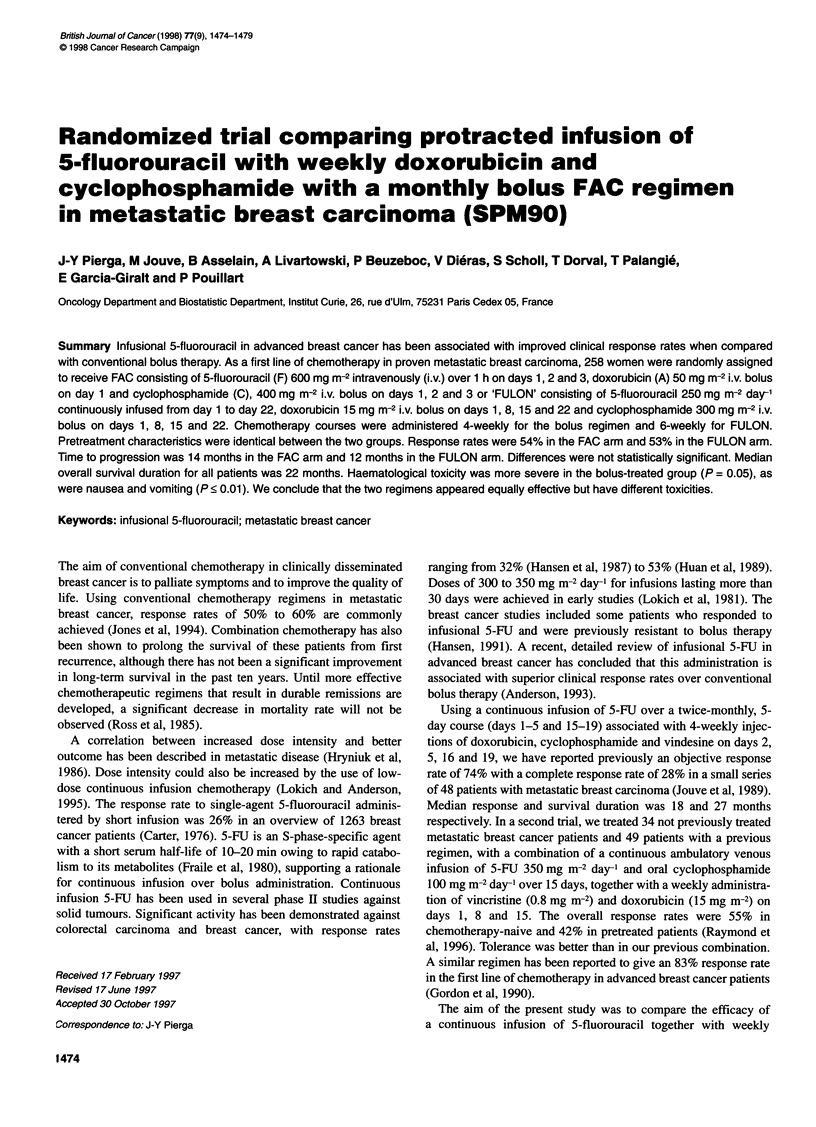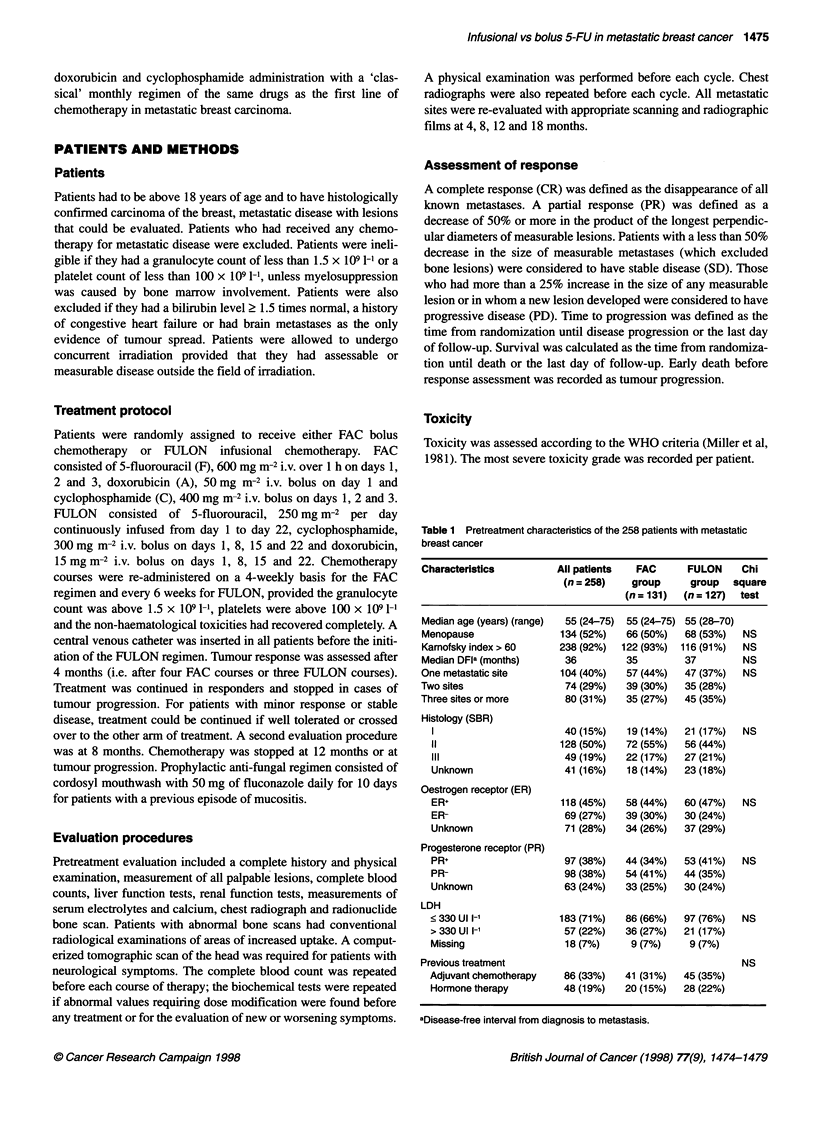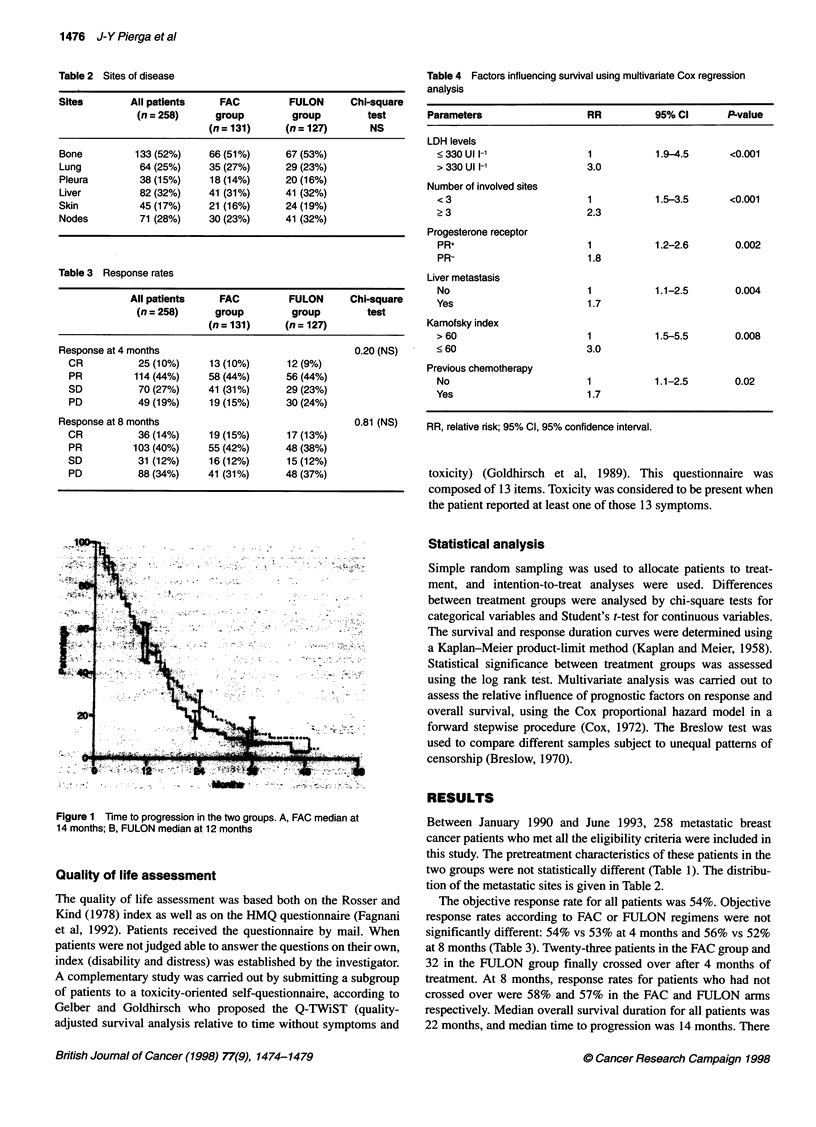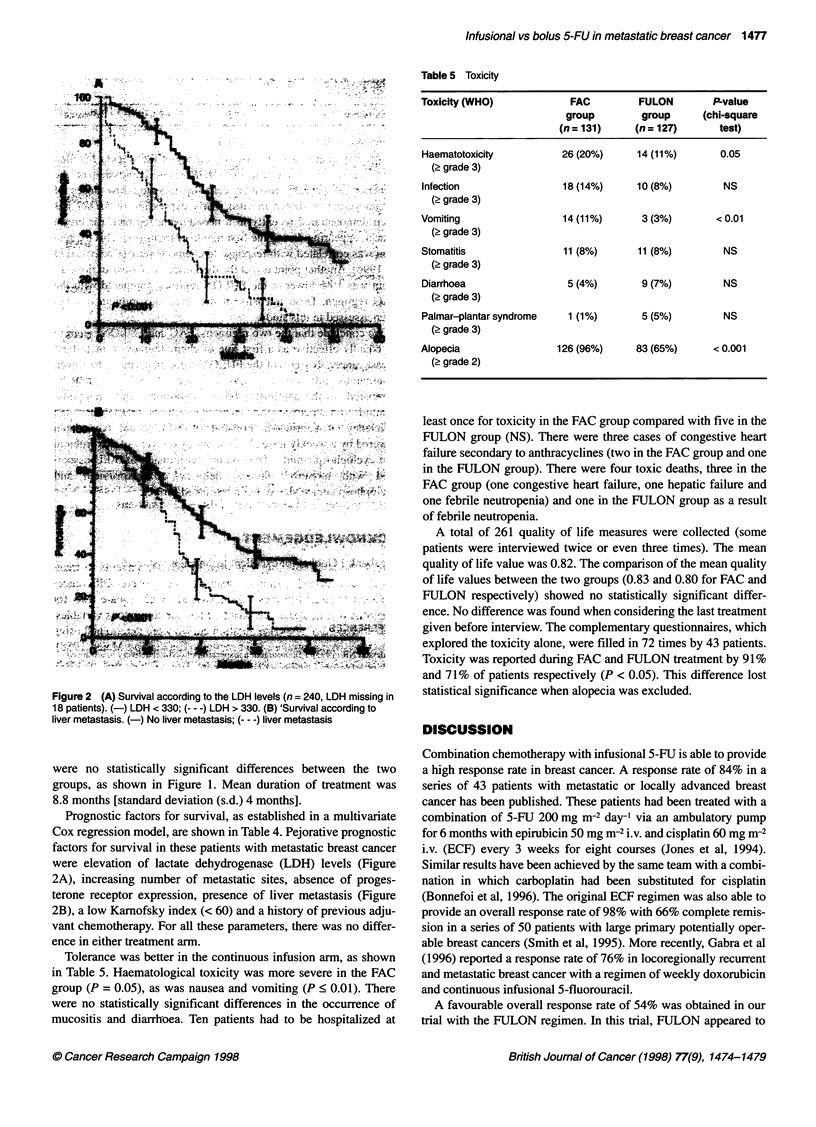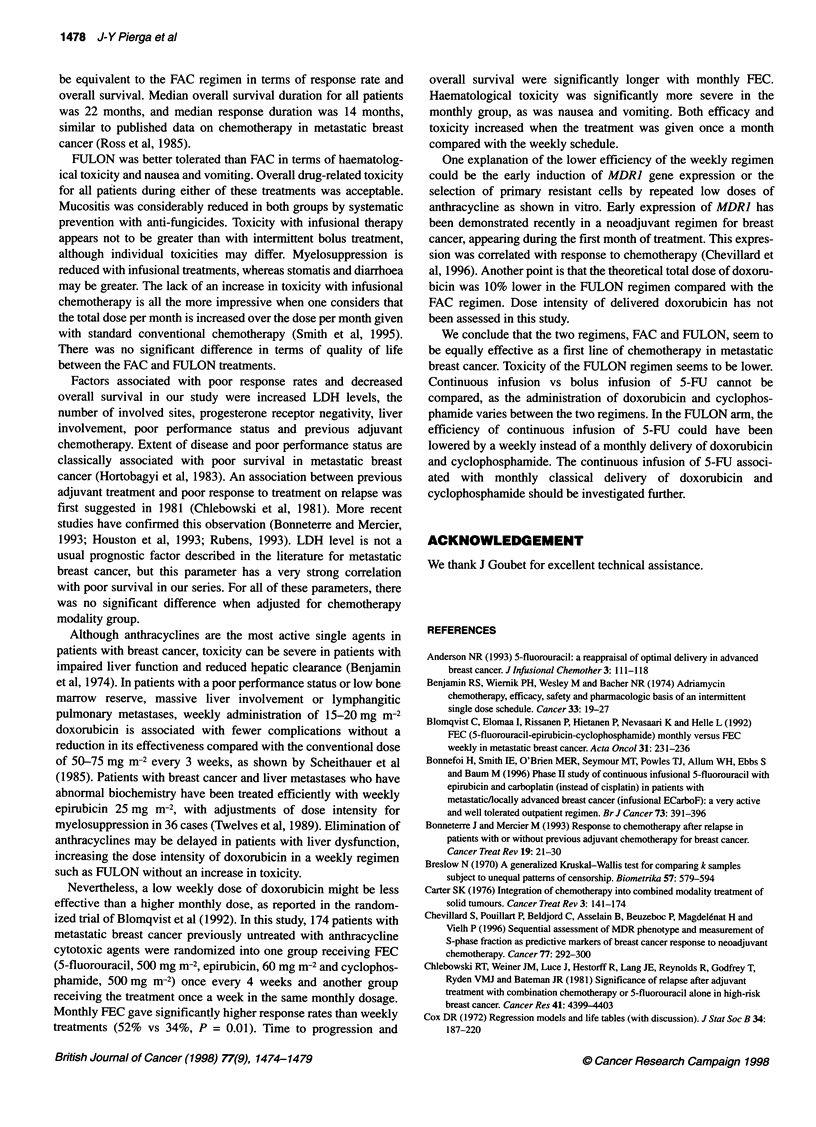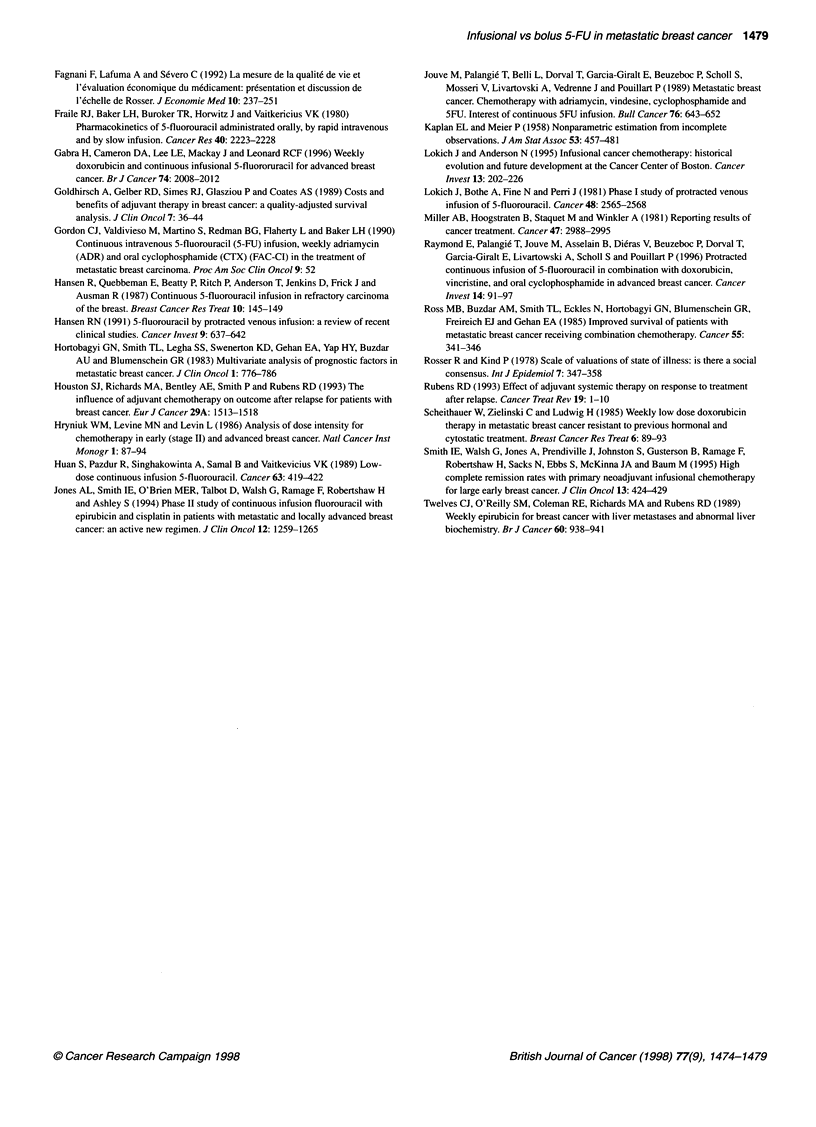# Randomized trial comparing protracted infusion of 5-fluorouracil with weekly doxorubicin and cyclophosphamide with a monthly bolus FAC regimen in metastatic breast carcinoma (SPM90).

**DOI:** 10.1038/bjc.1998.242

**Published:** 1998-05

**Authors:** J. Y. Pierga, M. Jouve, B. Asselain, A. Livartowski, P. Beuzeboc, V. DiÃ©ras, S. Scholl, T. Dorval, T. PalangiÃ©, E. Garcia-Giralt, P. Pouillart

**Affiliations:** Oncology Department, Institut Curie, Paris, France.

## Abstract

Infusional 5-fluorouracil in advanced breast cancer has been associated with improved clinical response rates when compared with conventional bolus therapy. As a first line of chemotherapy in proven metastatic breast carcinoma, 258 women were randomly assigned to receive FAC consisting of 5-fluorouracil (F) 600 mg m(-2) intravenously (i.v.) over 1 h on days 1, 2 and 3, doxorubicin (A) 50 mg m(-2) i.v. bolus on day 1 and cyclophosphamide (C), 400 mg m(-2) i.v. bolus on days 1, 2 and 3 or 'FULON' consisting of 5-fluorouracil 250 mg m(-2) day(-1) continuously infused from day 1 to day 22, doxorubicin 15 mg m(-2) i.v. bolus on days 1, 8, 15 and 22 and cyclophosphamide 300 mg m(-2) i.v. bolus on days 1, 8, 15 and 22. Chemotherapy courses were administered 4-weekly for the bolus regimen and 6-weekly for FULON. Pretreatment characteristics were identical between the two groups. Response rates were 54% in the FAC arm and 53% in the FULON arm. Time to progression was 14 months in the FAC arm and 12 months in the FULON arm. Differences were not statistically significant. Median overall survival duration for all patients was 22 months. Haematological toxicity was more severe in the bolus-treated group (P = 0.05), as were nausea and vomiting (P < or = 0.01). We conclude that the two regimens appeared equally effective but have different toxicities.


British Journal of Cancer (1998) 77(9), 1474–1479
© 1998 Cancer Research Campaign

### Randomized trial comparing protracted infusion of 5-fluorouracil with weekly doxorubicin and cyclophosphamide with a monthly bolus FAC regimen in metastatic breast carcinoma (SPM90)

**J-Y Pierga, M Jouve, B Asselain, A Livartowski, P Beuzeboc, V Diéras, S Scholl, T Dorval, T Palangié, E Garcia-Giralt and P Pouillart**

Oncology Department and Biostatistic Department, Institut Curie, 26, rue d'Ulm, 75231 Paris Cedex 05, France

**Summary** Infusional 5-fluorouracil in advanced breast cancer has been associated with improved clinical response rates when compared with conventional bolus therapy. As a first line of chemotherapy in proven metastatic breast carcinoma, 258 women were randomly assigned to receive FAC consisting of 5-fluorouracil (F) 600 mg m$^{-2}$ intravenously (i.v.) over 1 h on days 1, 2 and 3, doxorubicin (A) 50 mg m$^{-2}$ i.v. bolus on day 1 and cyclophosphamide (C), 400 mg m$^{-2}$ i.v. bolus on days 1, 2 and 3 or 'FULON' consisting of 5-fluorouracil 250 mg m$^{-2}$ day$^{-1}$ continuously infused from day 1 to day 22, doxorubicin 15 mg m$^{-2}$ i.v. bolus on days 1, 8, 15 and 22 and cyclophosphamide 300 mg m$^{-2}$ i.v. bolus on days 1, 8, 15 and 22. Chemotherapy courses were administered 4-weekly for the bolus regimen and 6-weekly for FULON. Pretreatment characteristics were identical between the two groups. Response rates were 54% in the FAC arm and 53% in the FULON arm. Time to progression was 14 months in the FAC arm and 12 months in the FULON arm. Differences were not statistically significant. Median overall survival duration for all patients was 22 months. Haematological toxicity was more severe in the bolus-treated group ($P = 0.05$), as were nausea and vomiting ($P \leq 0.01$). We conclude that the two regimens appeared equally effective but have different toxicities.

**Keywords:** infusional 5-fluorouracil; metastatic breast cancer

The aim of conventional chemotherapy in clinically disseminated breast cancer is to palliate symptoms and to improve the quality of life. Using conventional chemotherapy regimens in metastatic breast cancer, response rates of 50% to 60% are commonly achieved (Jones et al, 1994). Combination chemotherapy has also been shown to prolong the survival of these patients from first recurrence, although there has not been a significant improvement in long-term survival in the past ten years. Until more effective chemotherapeutic regimens that result in durable remissions are developed, a significant decrease in mortality rate will not be observed (Ross et al, 1985).

A correlation between increased dose intensity and better outcome has been described in metastatic disease (Hryniuk et al, 1986). Dose intensity could also be increased by the use of low-dose continuous infusion chemotherapy (Lokich and Anderson, 1995). The response rate to single-agent 5-fluorouracil administered by short infusion was 26% in an overview of 1263 breast cancer patients (Carter, 1976). 5-FU is an S-phase-specific agent with a short serum half-life of 10–20 min owing to rapid catabolism to its metabolites (Fraile et al, 1980), supporting a rationale for continuous infusion over bolus administration. Continuous infusion 5-FU has been used in several phase II studies against solid tumours. Significant activity has been demonstrated against colorectal carcinoma and breast cancer, with response rates ranging from 32% (Hansen et al, 1987) to 53% (Huan et al, 1989). Doses of 300 to 350 mg m$^{-2}$ day$^{-1}$ for infusions lasting more than 30 days were achieved in early studies (Lokich et al, 1981). The breast cancer studies included some patients who responded to infusional 5-FU and were previously resistant to bolus therapy (Hansen, 1991). A recent, detailed review of infusional 5-FU in advanced breast cancer has concluded that this administration is associated with superior clinical response rates over conventional bolus therapy (Anderson, 1993).

Using a continuous infusion of 5-FU over a twice-monthly, 5-day course (days 1–5 and 15–19) associated with 4-weekly injections of doxorubicin, cyclophosphamide and vindesine on days 2, 5, 16 and 19, we have reported previously an objective response rate of 74% with a complete response rate of 28% in a small series of 48 patients with metastatic breast carcinoma (Jouve et al, 1989). Median response and survival duration was 18 and 27 months respectively. In a second trial, we treated 34 not previously treated metastatic breast cancer patients and 49 patients with a previous regimen, with a combination of a continuous ambulatory venous infusion of 5-FU 350 mg m$^{-2}$ day$^{-1}$ and oral cyclophosphamide 100 mg m$^{-2}$ day$^{-1}$ over 15 days, together with a weekly administration of vincristine (0.8 mg m$^{-2}$) and doxorubicin (15 mg m$^{-2}$) on days 1, 8 and 15. The overall response rates were 55% in chemotherapy-naive and 42% in pretreated patients (Raymond et al, 1996). Tolerance was better than in our previous combination. A similar regimen has been reported to give an 83% response rate in the first line of chemotherapy in advanced breast cancer patients (Gordon et al, 1990).

The aim of the present study was to compare the efficacy of a continuous infusion of 5-fluorouracil together with weekly

*Received 17 February 1997*
*Revised 17 June 1997*
*Accepted 30 October 1997*

*Correspondence to:* J-Y Pierga doxorubicin and cyclophosphamide administration with a 'classical' monthly regimen of the same drugs as the first line of chemotherapy in metastatic breast carcinoma.

### PATIENTS AND METHODS

### Patients

Patients had to be above 18 years of age and to have histologically confirmed carcinoma of the breast, metastatic disease with lesions that could be evaluated. Patients who had received any chemotherapy for metastatic disease were excluded. Patients were ineligible if they had a granulocyte count of less than $1.5 \times 10^9\ l^{-1}$ or a platelet count of less than $100 \times 10^9\ l^{-1}$, unless myelosuppression was caused by bone marrow involvement. Patients were also excluded if they had a bilirubin level ≥ 1.5 times normal, a history of congestive heart failure or had brain metastases as the only evidence of tumour spread. Patients were allowed to undergo concurrent irradiation provided that they had assessable or measurable disease outside the field of irradiation.

### Treatment protocol

Patients were randomly assigned to receive either FAC bolus chemotherapy or FULON infusional chemotherapy. FAC consisted of 5-fluorouracil (F), 600 mg $m^{-2}$ i.v. over 1 h on days 1, 2 and 3, doxorubicin (A), 50 mg $m^{-2}$ i.v. bolus on day 1 and cyclophosphamide (C), 400 mg $m^{-2}$ i.v. bolus on days 1, 2 and 3. FULON consisted of 5-fluorouracil, 250 mg $m^{-2}$ per day continuously infused from day 1 to day 22, cyclophosphamide, 300 mg $m^{-2}$ i.v. bolus on days 1, 8, 15 and 22 and doxorubicin, 15 mg $m^{-2}$ i.v. bolus on days 1, 8, 15 and 22. Chemotherapy courses were re-administered on a 4-weekly basis for the FAC regimen and every 6 weeks for FULON, provided the granulocyte count was above $1.5 \times 10^9\ l^{-1}$, platelets were above $100 \times 10^9\ l^{-1}$ and the non-haematological toxicities had recovered completely. A central venous catheter was inserted in all patients before the initiation of the FULON regimen. Tumour response was assessed after 4 months (i.e. after four FAC courses or three FULON courses). Treatment was continued in responders and stopped in cases of tumour progression. For patients with minor response or stable disease, treatment could be continued if well tolerated or crossed over to the other arm of treatment. A second evaluation procedure was at 8 months. Chemotherapy was stopped at 12 months or at tumour progression. Prophylactic anti-fungal regimen consisted of cordosyl mouthwash with 50 mg of fluconazole daily for 10 days for patients with a previous episode of mucositis.

### Evaluation procedures

Pretreatment evaluation included a complete history and physical examination, measurement of all palpable lesions, complete blood counts, liver function tests, renal function tests, measurements of serum electrolytes and calcium, chest radiograph and radionuclide bone scan. Patients with abnormal bone scans had conventional radiological examinations of areas of increased uptake. A computerized tomographic scan of the head was required for patients with neurological symptoms. The complete blood count was repeated before each course of therapy; the biochemical tests were repeated if abnormal values requiring dose modification were found before any treatment or for the evaluation of new or worsening symptoms. A physical examination was performed before each cycle. Chest radiographs were also repeated before each cycle. All metastatic sites were re-evaluated with appropriate scanning and radiographic films at 4, 8, 12 and 18 months.

### Assessment of response

A complete response (CR) was defined as the disappearance of all known metastases. A partial response (PR) was defined as a decrease of 50% or more in the product of the longest perpendicular diameters of measurable lesions. Patients with a less than 50% decrease in the size of measurable metastases (which excluded bone lesions) were considered to have stable disease (SD). Those who had more than a 25% increase in the size of any measurable lesion or in whom a new lesion developed were considered to have progressive disease (PD). Time to progression was defined as the time from randomization until disease progression or the last day of follow-up. Survival was calculated as the time from randomization until death or the last day of follow-up. Early death before response assessment was recorded as tumour progression.

### Toxicity

Toxicity was assessed according to the WHO criteria (Miller et al, 1981). The most severe toxicity grade was recorded per patient.

**Table 1** Pretreatment characteristics of the 258 patients with metastatic breast cancer

| Characteristics | All patients (*n* = 258) | FAC group (*n* = 131) | FULON group (*n* = 127) | Chi square test |
|---|---|---|---|---|
| Median age (years) (range) | 55 (24–75) | 55 (24–75) | 55 (28–70) | |
| Menopause | 134 (52%) | 66 (50%) | 68 (53%) | NS |
| Karnofsky index > 60 | 238 (92%) | 122 (93%) | 116 (91%) | NS |
| Median DFI[a] (months) | 36 | 35 | 37 | NS |
| One metastatic site | 104 (40%) | 57 (44%) | 47 (37%) | NS |
| Two sites | 74 (29%) | 39 (30%) | 35 (28%) | |
| Three sites or more | 80 (31%) | 35 (27%) | 45 (35%) | |
| Histology (SBR) | | | | |
| I | 40 (15%) | 19 (14%) | 21 (17%) | NS |
| II | 128 (50%) | 72 (55%) | 56 (44%) | |
| III | 49 (19%) | 22 (17%) | 27 (21%) | |
| Unknown | 41 (16%) | 18 (14%) | 23 (18%) | |
| Oestrogen receptor (ER) | | | | |
| $ER^+$ | 118 (45%) | 58 (44%) | 60 (47%) | NS |
| $ER^-$ | 69 (27%) | 39 (30%) | 30 (24%) | |
| Unknown | 71 (28%) | 34 (26%) | 37 (29%) | |
| Progesterone receptor (PR) | | | | |
| $PR^+$ | 97 (38%) | 44 (34%) | 53 (41%) | NS |
| $PR^-$ | 98 (38%) | 54 (41%) | 44 (35%) | |
| Unknown | 63 (24%) | 33 (25%) | 30 (24%) | |
| LDH | | | | |
| ≤ 330 UI $l^{-1}$ | 183 (71%) | 86 (66%) | 97 (76%) | NS |
| > 330 UI $l^{-1}$ | 57 (22%) | 36 (27%) | 21 (17%) | |
| Missing | 18 (7%) | 9 (7%) | 9 (7%) | |
| Previous treatment | | | | NS |
| Adjuvant chemotherapy | 86 (33%) | 41 (31%) | 45 (35%) | |
| Hormone therapy | 48 (19%) | 20 (15%) | 28 (22%) | |

[a]Disease-free interval from diagnosis to metastasis.

© Cancer Research Campaign 1998

**Table 2** Sites of disease

| Sites | All patients (n = 258) | FAC group (n = 131) | FULON group (n = 127) | Chi-square test NS |
|---|---|---|---|---|
| Bone | 133 (52%) | 66 (51%) | 67 (53%) | |
| Lung | 64 (25%) | 35 (27%) | 29 (23%) | |
| Pleura | 38 (15%) | 18 (14%) | 20 (16%) | |
| Liver | 82 (32%) | 41 (31%) | 41 (32%) | |
| Skin | 45 (17%) | 21 (16%) | 24 (19%) | |
| Nodes | 71 (28%) | 30 (23%) | 41 (32%) | |

**Table 3** Response rates

| | All patients (n = 258) | FAC group (n = 131) | FULON group (n = 127) | Chi-square test |
|---|---|---|---|---|
| Response at 4 months | | | | 0.20 (NS) |
| CR | 25 (10%) | 13 (10%) | 12 (9%) | |
| PR | 114 (44%) | 58 (44%) | 56 (44%) | |
| SD | 70 (27%) | 41 (31%) | 29 (23%) | |
| PD | 49 (19%) | 19 (15%) | 30 (24%) | |
| Response at 8 months | | | | 0.81 (NS) |
| CR | 36 (14%) | 19 (15%) | 17 (13%) | |
| PR | 103 (40%) | 55 (42%) | 48 (38%) | |
| SD | 31 (12%) | 16 (12%) | 15 (12%) | |
| PD | 88 (34%) | 41 (31%) | 48 (37%) | |

**Figure 1** Time to progression in the two groups. A, FAC median at 14 months; B, FULON median at 12 months

### Quality of life assessment

The quality of life assessment was based both on the Rosser and Kind (1978) index as well as on the HMQ questionnaire (Fagnani et al, 1992). Patients received the questionnaire by mail. When patients were not judged able to answer the questions on their own, index (disability and distress) was established by the investigator. A complementary study was carried out by submitting a subgroup of patients to a toxicity-oriented self-questionnaire, according to Gelber and Goldhirsch who proposed the Q-TWiST (quality-adjusted survival analysis relative to time without symptoms and toxicity) (Goldhirsch et al, 1989). This questionnaire was composed of 13 items. Toxicity was considered to be present when the patient reported at least one of those 13 symptoms.

**Table 4** Factors influencing survival using multivariate Cox regression analysis

| Parameters | RR | 95% CI | P-value |
|---|---|---|---|
| LDH levels | | | |
| ≤ 330 UI $l^{-1}$ | 1 | 1.9–4.5 | <0.001 |
| > 330 UI $l^{-1}$ | 3.0 | | |
| Number of involved sites | | | |
| < 3 | 1 | 1.5–3.5 | <0.001 |
| ≥ 3 | 2.3 | | |
| Progesterone receptor | | | |
| $PR^+$ | 1 | 1.2–2.6 | 0.002 |
| $PR^-$ | 1.8 | | |
| Liver metastasis | | | |
| No | 1 | 1.1–2.5 | 0.004 |
| Yes | 1.7 | | |
| Karnofsky index | | | |
| > 60 | 1 | 1.5–5.5 | 0.008 |
| ≤ 60 | 3.0 | | |
| Previous chemotherapy | | | |
| No | 1 | 1.1–2.5 | 0.02 |
| Yes | 1.7 | | |

RR, relative risk; 95% CI, 95% confidence interval.

### Statistical analysis

Simple random sampling was used to allocate patients to treatment, and intention-to-treat analyses were used. Differences between treatment groups were analysed by chi-square tests for categorical variables and Student's t-test for continuous variables. The survival and response duration curves were determined using a Kaplan–Meier product-limit method (Kaplan and Meier, 1958). Statistical significance between treatment groups was assessed using the log rank test. Multivariate analysis was carried out to assess the relative influence of prognostic factors on response and overall survival, using the Cox proportional hazard model in a forward stepwise procedure (Cox, 1972). The Breslow test was used to compare different samples subject to unequal patterns of censorship (Breslow, 1970).

### RESULTS

Between January 1990 and June 1993, 258 metastatic breast cancer patients who met all the eligibility criteria were included in this study. The pretreatment characteristics of these patients in the two groups were not statistically different (Table 1). The distribution of the metastatic sites is given in Table 2.

The objective response rate for all patients was 54%. Objective response rates according to FAC or FULON regimens were not significantly different: 54% vs 53% at 4 months and 56% vs 52% at 8 months (Table 3). Twenty-three patients in the FAC group and 32 in the FULON group finally crossed over after 4 months of treatment. At 8 months, response rates for patients who had not crossed over were 58% and 57% in the FAC and FULON arms respectively. Median overall survival duration for all patients was 22 months, and median time to progression was 14 months. There

© Cancer Research Campaign 1998

**Figure 2** **(A)** Survival according to the LDH levels (*n* = 240, LDH missing in 18 patients). (—) LDH < 330; (- - -) LDH > 330. **(B)** 'Survival according to liver metastasis. (—) No liver metastasis; (- - -) liver metastasis were no statistically significant differences between the two groups, as shown in Figure 1. Mean duration of treatment was 8.8 months [standard deviation (s.d.) 4 months].

Prognostic factors for survival, as established in a multivariate Cox regression model, are shown in Table 4. Pejorative prognostic factors for survival in these patients with metastatic breast cancer were elevation of lactate dehydrogenase (LDH) levels (Figure 2A), increasing number of metastatic sites, absence of progesterone receptor expression, presence of liver metastasis (Figure 2B), a low Karnofsky index (< 60) and a history of previous adjuvant chemotherapy. For all these parameters, there was no difference in either treatment arm.

Tolerance was better in the continuous infusion arm, as shown in Table 5. Haematological toxicity was more severe in the FAC group ($P = 0.05$), as was nausea and vomiting ($P \leq 0.01$). There were no statistically significant differences in the occurrence of mucositis and diarrhoea. Ten patients had to be hospitalized at

**Table 5** Toxicity

| Toxicity (WHO) | FAC group (*n* = 131) | FULON group (*n* = 127) | *P*-value (chi-square test) |
|---|---|---|---|
| Haematotoxicity (≥ grade 3) | 26 (20%) | 14 (11%) | 0.05 |
| Infection (≥ grade 3) | 18 (14%) | 10 (8%) | NS |
| Vomiting (≥ grade 3) | 14 (11%) | 3 (3%) | < 0.01 |
| Stomatitis (≥ grade 3) | 11 (8%) | 11 (8%) | NS |
| Diarrhoea (≥ grade 3) | 5 (4%) | 9 (7%) | NS |
| Palmar–plantar syndrome (≥ grade 3) | 1 (1%) | 5 (5%) | NS |
| Alopecia (≥ grade 2) | 126 (96%) | 83 (65%) | < 0.001 |

least once for toxicity in the FAC group compared with five in the FULON group (NS). There were three cases of congestive heart failure secondary to anthracyclines (two in the FAC group and one in the FULON group). There were four toxic deaths, three in the FAC group (one congestive heart failure, one hepatic failure and one febrile neutropenia) and one in the FULON group as a result of febrile neutropenia.

A total of 261 quality of life measures were collected (some patients were interviewed twice or even three times). The mean quality of life value was 0.82. The comparison of the mean quality of life values between the two groups (0.83 and 0.80 for FAC and FULON respectively) showed no statistically significant difference. No difference was found when considering the last treatment given before interview. The complementary questionnaires, which explored the toxicity alone, were filled in 72 times by 43 patients. Toxicity was reported during FAC and FULON treatment by 91% and 71% of patients respectively ($P < 0.05$). This difference lost statistical significance when alopecia was excluded.

### DISCUSSION

Combination chemotherapy with infusional 5-FU is able to provide a high response rate in breast cancer. A response rate of 84% in a series of 43 patients with metastatic or locally advanced breast cancer has been published. These patients had been treated with a combination of 5-FU 200 mg $m^{-2}$ $day^{-1}$ via an ambulatory pump for 6 months with epirubicin 50 mg $m^{-2}$ i.v. and cisplatin 60 mg $m^{-2}$ i.v. (ECF) every 3 weeks for eight courses (Jones et al, 1994). Similar results have been achieved by the same team with a combination in which carboplatin had been substituted for cisplatin (Bonnefoi et al, 1996). The original ECF regimen was also able to provide an overall response rate of 98% with 66% complete remission in a series of 50 patients with large primary potentially operable breast cancers (Smith et al, 1995). More recently, Gabra et al (1996) reported a response rate of 76% in locoregionally recurrent and metastatic breast cancer with a regimen of weekly doxorubicin and continuous infusional 5-fluorouracil.

A favourable overall response rate of 54% was obtained in our trial with the FULON regimen. In this trial, FULON appeared to

© Cancer Research Campaign 1998

be equivalent to the FAC regimen in terms of response rate and overall survival. Median overall survival duration for all patients was 22 months, and median response duration was 14 months, similar to published data on chemotherapy in metastatic breast cancer (Ross et al, 1985).

FULON was better tolerated than FAC in terms of haematological toxicity and nausea and vomiting. Overall drug-related toxicity for all patients during either of these treatments was acceptable. Mucositis was considerably reduced in both groups by systematic prevention with anti-fungicides. Toxicity with infusional therapy appears not to be greater than with intermittent bolus treatment, although individual toxicities may differ. Myelosuppression is reduced with infusional treatments, whereas stomatis and diarrhoea may be greater. The lack of an increase in toxicity with infusional chemotherapy is all the more impressive when one considers that the total dose per month is increased over the dose per month given with standard conventional chemotherapy (Smith et al, 1995). There was no significant difference in terms of quality of life between the FAC and FULON treatments.

Factors associated with poor response rates and decreased overall survival in our study were increased LDH levels, the number of involved sites, progesterone receptor negativity, liver involvement, poor performance status and previous adjuvant chemotherapy. Extent of disease and poor performance status are classically associated with poor survival in metastatic breast cancer (Hortobagyi et al, 1983). An association between previous adjuvant treatment and poor response to treatment on relapse was first suggested in 1981 (Chlebowski et al, 1981). More recent studies have confirmed this observation (Bonneterre and Mercier, 1993; Houston et al, 1993; Rubens, 1993). LDH level is not a usual prognostic factor described in the literature for metastatic breast cancer, but this parameter has a very strong correlation with poor survival in our series. For all of these parameters, there was no significant difference when adjusted for chemotherapy modality group.

Although anthracyclines are the most active single agents in patients with breast cancer, toxicity can be severe in patients with impaired liver function and reduced hepatic clearance (Benjamin et al, 1974). In patients with a poor performance status or low bone marrow reserve, massive liver involvement or lymphangitic pulmonary metastases, weekly administration of 15–20 mg $m^{-2}$ doxorubicin is associated with fewer complications without a reduction in its effectiveness compared with the conventional dose of 50–75 mg $m^{-2}$ every 3 weeks, as shown by Scheithauer et al (1985). Patients with breast cancer and liver metastases who have abnormal biochemistry have been treated efficiently with weekly epirubicin 25 mg $m^{-2}$, with adjustments of dose intensity for myelosuppression in 36 cases (Twelves et al, 1989). Elimination of anthracyclines may be delayed in patients with liver dysfunction, increasing the dose intensity of doxorubicin in a weekly regimen such as FULON without an increase in toxicity.

Nevertheless, a low weekly dose of doxorubicin might be less effective than a higher monthly dose, as reported in the randomized trial of Blomqvist et al (1992). In this study, 174 patients with metastatic breast cancer previously untreated with anthracycline cytotoxic agents were randomized into one group receiving FEC (5-fluorouracil, 500 mg $m^{-2}$, epirubicin, 60 mg $m^{-2}$ and cyclophosphamide, 500 mg $m^{-2}$) once every 4 weeks and another group receiving the treatment once a week in the same monthly dosage. Monthly FEC gave significantly higher response rates than weekly treatments (52% vs 34%, $P$ = 0.01). Time to progression and overall survival were significantly longer with monthly FEC. Haematological toxicity was significantly more severe in the monthly group, as was nausea and vomiting. Both efficacy and toxicity increased when the treatment was given once a month compared with the weekly schedule.

One explanation of the lower efficiency of the weekly regimen could be the early induction of *MDR1* gene expression or the selection of primary resistant cells by repeated low doses of anthracycline as shown in vitro. Early expression of *MDR1* has been demonstrated recently in a neoadjuvant regimen for breast cancer, appearing during the first month of treatment. This expression was correlated with response to chemotherapy (Chevillard et al, 1996). Another point is that the theoretical total dose of doxorubicin was 10% lower in the FULON regimen compared with the FAC regimen. Dose intensity of delivered doxorubicin has not been assessed in this study.

We conclude that the two regimens, FAC and FULON, seem to be equally effective as a first line of chemotherapy in metastatic breast cancer. Toxicity of the FULON regimen seems to be lower. Continuous infusion vs bolus infusion of 5-FU cannot be compared, as the administration of doxorubicin and cyclophosphamide varies between the two regimens. In the FULON arm, the efficiency of continuous infusion of 5-FU could have been lowered by a weekly instead of a monthly delivery of doxorubicin and cyclophosphamide. The continuous infusion of 5-FU associated with monthly classical delivery of doxorubicin and cyclophosphamide should be investigated further.

### ACKNOWLEDGEMENT

We thank J Goubet for excellent technical assistance.

### REFERENCES

Anderson NR (1993) 5-fluorouracil: a reappraisal of optimal delivery in advanced breast cancer. *J Infusional Chemother* **3**: 111–118

Benjamin RS, Wiernik PH, Wesley M and Bacher NR (1974) Adriamycin chemotherapy, efficacy, safety and pharmacologic basis of an intermittent single dose schedule. *Cancer* **33**: 19–27

Blomqvist C, Elomaa I, Rissanen P, Hietanen P, Nevasaari K and Helle L (1992) FEC (5-fluorouracil-epirubicin-cyclophosphamide) monthly versus FEC weekly in metastatic breast cancer. *Acta Oncol* **31**: 231–236

Bonnefoi H, Smith IE, O'Brien MER, Seymour MT, Powles TJ, Allum WH, Ebbs S and Baum M (1996) Phase II study of continuous infusional 5-fluorouracil with epirubicin and carboplatin (instead of cisplatin) in patients with metastatic/locally advanced breast cancer (infusional ECarboF): a very active and well tolerated outpatient regimen. *Br J Cancer* **73**: 391–396

Bonneterre J and Mercier M (1993) Response to chemotherapy after relapse in patients with or without previous adjuvant chemotherapy for breast cancer. *Cancer Treat Rev* **19**: 21–30

Breslow N (1970) A generalized Kruskal–Wallis test for comparing *k* samples subject to unequal patterns of censorship. *Biometrika* **57**: 579–594

Carter SK (1976) Integration of chemotherapy into combined modality treatment of solid tumours. *Cancer Treat Rev* **3**: 141–174

Chevillard S, Pouillart P, Beldjord C, Asselain B, Beuzeboc P, Magdelénat H and Vielh P (1996) Sequential assessment of MDR phenotype and measurement of S-phase fraction as predictive markers of breast cancer response to neoadjuvant chemotherapy. *Cancer* **77**: 292–300

Chlebowski RT, Weiner JM, Luce J, Hestorff R, Lang JE, Reynolds R, Godfrey T, Ryden VMJ and Bateman JR (1981) Significance of relapse after adjuvant treatment with combination chemotherapy or 5-fluorouracil alone in high-risk breast cancer. *Cancer Res* **41**: 4399–4403

Cox DR (1972) Regression models and life tables (with discussion). *J Stat Soc B* **34**: 187–220

© Cancer Research Campaign 1998

Fagnani F, Lafuma A and Sévero C (1992) La mesure de la qualité de vie et l'évaluation économique du médicament: présentation et discussion de l'échelle de Rosser. *J Economie Med* **10**: 237–251

Fraile RJ, Baker LH, Buroker TR, Horwitz J and Vaitkericius VK (1980) Pharmacokinetics of 5-fluorouracil administrated orally, by rapid intravenous and by slow infusion. *Cancer Res* **40**: 2223–2228

Gabra H, Cameron DA, Lee LE, Mackay J and Leonard RCF (1996) Weekly doxorubicin and continuous infusional 5-fluororuracil for advanced breast cancer. *Br J Cancer* **74**: 2008–2012

Goldhirsch A, Gelber RD, Simes RJ, Glasziou P and Coates AS (1989) Costs and benefits of adjuvant therapy in breast cancer: a quality-adjusted survival analysis. *J Clin Oncol* **7**: 36–44

Gordon CJ, Valdivieso M, Martino S, Redman BG, Flaherty L and Baker LH (1990) Continuous intravenous 5-fluorouracil (5-FU) infusion, weekly adriamycin (ADR) and oral cyclophosphamide (CTX) (FAC-CI) in the treatment of metastatic breast carcinoma. *Proc Am Soc Clin Oncol* **9**: 52

Hansen R, Quebbeman E, Beatty P, Ritch P, Anderson T, Jenkins D, Frick J and Ausman R (1987) Continuous 5-fluorouracil infusion in refractory carcinoma of the breast. *Breast Cancer Res Treat* **10**: 145–149

Hansen RN (1991) 5-fluorouracil by protracted venous infusion: a review of recent clinical studies. *Cancer Invest* **9**: 637–642

Hortobagyi GN, Smith TL, Legha SS, Swenerton KD, Gehan EA, Yap HY, Buzdar AU and Blumenschein GR (1983) Multivariate analysis of prognostic factors in metastatic breast cancer. *J Clin Oncol* **1**: 776–786

Houston SJ, Richards MA, Bentley AE, Smith P and Rubens RD (1993) The influence of adjuvant chemotherapy on outcome after relapse for patients with breast cancer. *Eur J Cancer* **29A**: 1513–1518

Hryniuk WM, Levine MN and Levin L (1986) Analysis of dose intensity for chemotherapy in early (stage II) and advanced breast cancer. *Natl Cancer Inst Monogr* **1**: 87–94

Huan S, Pazdur R, Singhakowinta A, Samal B and Vaitkevicius VK (1989) Low-dose continuous infusion 5-fluorouracil. *Cancer* **63**: 419–422

Jones AL, Smith IE, O'Brien MER, Talbot D, Walsh G, Ramage F, Robertshaw H and Ashley S (1994) Phase II study of continuous infusion fluorouracil with epirubicin and cisplatin in patients with metastatic and locally advanced breast cancer: an active new regimen. *J Clin Oncol* **12**: 1259–1265

Jouve M, Palangié T, Belli L, Dorval T, Garcia-Giralt E, Beuzeboc P, Scholl S, Mosseri V, Livartovski A, Vedrenne J and Pouillart P (1989) Metastatic breast cancer. Chemotherapy with adriamycin, vindesine, cyclophosphamide and 5FU. Interest of continuous 5FU infusion. *Bull Cancer* **76**: 643–652

Kaplan EL and Meier P (1958) Nonparametric estimation from incomplete observations. *J Am Stat Assoc* **53**: 457–481

Lokich J and Anderson N (1995) Infusional cancer chemotherapy: historical evolution and future development at the Cancer Center of Boston. *Cancer Invest* **13**: 202–226

Lokich J, Bothe A, Fine N and Perri J (1981) Phase I study of protracted venous infusion of 5-fluorouracil. *Cancer* **48**: 2565–2568

Miller AB, Hoogstraten B, Staquet M and Winkler A (1981) Reporting results of cancer treatment. *Cancer* **47**: 2988–2995

Raymond E, Palangié T, Jouve M, Asselain B, Diéras V, Beuzeboc P, Dorval T, Garcia-Giralt E, Livartowski A, Scholl S and Pouillart P (1996) Protracted continuous infusion of 5-fluorouracil in combination with doxorubicin, vincristine, and oral cyclophosphamide in advanced breast cancer. *Cancer Invest* **14**: 91–97

Ross MB, Buzdar AM, Smith TL, Eckles N, Hortobagyi GN, Blumenschein GR, Freireich EJ and Gehan EA (1985) Improved survival of patients with metastatic breast cancer receiving combination chemotherapy. *Cancer* **55**: 341–346

Rosser R and Kind P (1978) Scale of valuations of state of illness: is there a social consensus. *Int J Epidemiol* **7**: 347–358

Rubens RD (1993) Effect of adjuvant systemic therapy on response to treatment after relapse. *Cancer Treat Rev* **19**: 1–10

Scheithauer W, Zielinski C and Ludwig H (1985) Weekly low dose doxorubicin therapy in metastatic breast cancer resistant to previous hormonal and cytostatic treatment. *Breast Cancer Res Treat* **6**: 89–93

Smith IE, Walsh G, Jones A, Prendiville J, Johnston S, Gusterson B, Ramage F, Robertshaw H, Sacks N, Ebbs S, McKinna JA and Baum M (1995) High complete remission rates with primary neoadjuvant infusional chemotherapy for large early breast cancer. *J Clin Oncol* **13**: 424–429

Twelves CJ, O'Reilly SM, Coleman RE, Richards MA and Rubens RD (1989) Weekly epirubicin for breast cancer with liver metastases and abnormal liver biochemistry. *Br J Cancer* **60**: 938–941

© Cancer Research Campaign 1998